# Isolated Liver Metastasis in Hürthle Cell Thyroid Cancer Treated with Microwave Ablation

**DOI:** 10.1155/2017/2790741

**Published:** 2017-01-09

**Authors:** Konstantinos Segkos, Carl Schmidt, Fadi Nabhan

**Affiliations:** ^1^Endocrinology, Diabetes and Metabolism, The Ohio State University Wexner Medical Center, 5th Floor McCampbell Hall, 1581 Dodd Drive, Columbus, OH 43210, USA; ^2^Surgical Oncology, The Ohio State University Wexner Medical Center, N-924 Doan Hall, 410 W. 10th Avenue, Columbus, OH 43210, USA

## Abstract

Hürthle cell thyroid cancer (HCTC) is a less common form of differentiated thyroid cancer. It rarely metastasizes to the liver, and when it does, the metastasis is almost never isolated. Here we report a 62-year-old male with widely invasive Hürthle cell thyroid cancer, who underwent total thyroidectomy and received adjuvant treatment with I-131 with posttreatment scan showing no evidence of metastatic disease. His thyroglobulin however continued to rise after that and eventually an isolated liver metastasis was identified. He underwent laparoscopic microwave ablation of the liver metastasis, with dramatic decline in thyroglobulin and no structural disease identified to date. This case highlights the rare occurrence of isolated liver metastasis from HCTC and also illustrates the utility of thermoablation as an alternative to surgical resection in the treatment of small isolated liver metastases from HCTC.

## 1. Introduction

Hürthle cell thyroid carcinoma (HCTC) accounts for 3% of all thyroid malignancies. If distant metastases develop, then the most common site is the lung, followed by bone, with other sites being much rarer [1]. When liver metastases are present, they are almost always multiple or diffuse and are usually accompanied by metastases at other sites. We present a rare case of HCTC, with an isolated liver metastasis, treated with intraoperative microwave ablation (MWA).

## 2. Case Presentation

A 62-year-old male presented with dysphagia for 6 months and a palpable neck mass. A neck ultrasound (US) showed a 5.3 cm solid hypoechoic mass. He underwent an ultrasound-guided thyroid fine needle aspiration (FNA). The cytology was suspicious but not diagnostic for anaplastic thyroid cancer. He underwent total thyroidectomy with final pathology demonstrating a 7.4 cm HCTC, with breached capsule, no extrathyroidal extension, and vascular space invasion (6 vessels). His postoperative thyroglobulin (Tg) level was at 40 ng/mL (Table 1). He received 152 mCi I-131 with recombinant TSH stimulation. A posttreatment scan only showed persistent radioiodine activity in the right thyroid bed.

**Figure 1:**
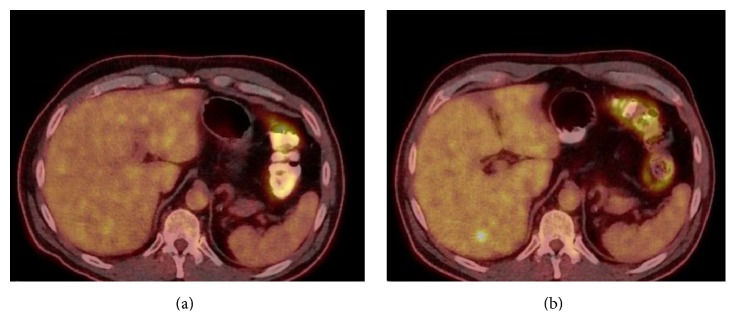
PET/CT at 4 months (a) and 8 months (b) after total thyroidectomy. There is new focal uptake within the posterior right lobe of the liver, measuring a maximum SUV of 5.4, consistent with metastatic disease. This was not evident on the initial PET/CT.

**Figure 2:**
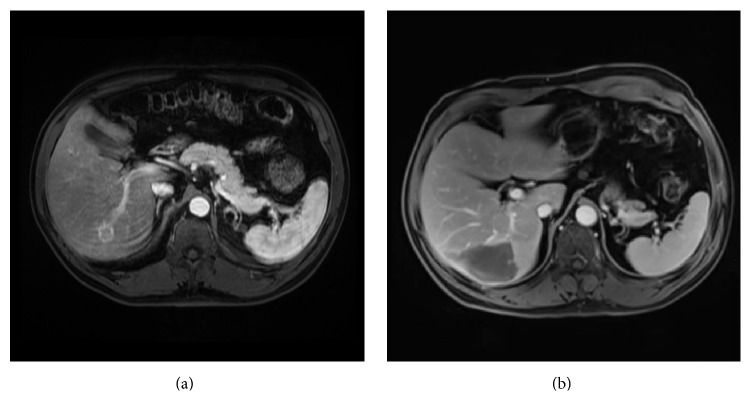
Abdominal MRI before microwave ablation shows a rounded lesion in liver segment 6, measuring 2.1 × 2.1 cm, which demonstrates T2 hyperintensity with heterogeneous internal enhancement or restricted diffusion on MRI (a). 2 months after microwave ablation of the liver, there is evidence of postoperative and postmicrowave changes in the liver, with no suspicious enhancement in the ablation bed to suggest definite residual or recurrent tumor, and no new focal liver lesions (b).

Over the following 7 months, his Tg gradually increased to 318.1 ng/mL (Table 1). A neck US, neck computed tomography (CT), chest CT, and brain magnetic resonance imaging (MRI) were unremarkable. A positron emission tomography-computed tomography (PET-CT) at 4 months postoperatively was unremarkable (Figure 1(a)). A CT abdomen and pelvis at 8 months postoperatively demonstrated a new isolated hypodense lesion in the posterior lobe of the liver. PET scan was repeated and this lesion was fluorodeoxyglucose (FDG) avid (Figure 1(b)), and it was also confirmed with an abdominal MRI (Figure 2(a)). Overall, the lesion was consistent with a metastatic deposit.

The metastasis was deep in the right lobe of the liver. In order to remove it surgically, it would have required a major open liver resection. Given the possibility that other metastases would arise in the future and in order to avoid the morbidity of this procedure, the patient underwent simultaneous laparoscopic core biopsy and MWA of the liver mass, with intraoperative ultrasound guidance. The liver biopsy confirmed carcinoma metastatic to the liver, compatible with thyroid gland origin.

One month later, Tg dropped to 0.6 ng/mL. Abdominal MRI did not reveal residual or recurrent tumor (Figure 2(b)). His Tg has slowly increased to 1.3 ng/mL at 9-month follow-up and 1.9 ng/mL at 12-month follow-up after the ablation of the liver metastasis (Table 1). The Tg antibodies have remained undetectable. Up to date, with 12-month follow-up, no evidence of structural disease has been found with negative neck ultrasound, neck and chest CT, and abdominal MRI.

## 3. Discussion

HCTC has traditionally been considered as a variant of follicular thyroid cancer (FTC) [1, 2]. However, other data suggest that it is a distinct thyroid malignancy and accounts for 3% of all thyroid malignancies [1, 3, 4]. Nagar et al. performed a retrospective review of the Surveillance, Epidemiology, and End Results (SEER) database and concluded that although in the past HCTC had worse prognosis than FTC, the survival rate of patients with HCTC has improved over the years and is now the same as the survival rate for FTC [5].

HCTC also has a greater propensity for distant metastases. A review of 108 patients with HCTC by Besic et al. identified 32 patients with distant metastatic disease either at presentation or during clinical follow-up. The most common site of distant metastases was the lung, followed by bone. Other sites were much less common, with liver being only one of them, and it was also accompanied by lung metastases [1]. This distribution of distant metastatic disease sites is similar to PTC and FTC [6]. Other metastatic sites in patients with differentiated thyroid cancer (DTC) are considered rare (frequency < 1%) and include brain, liver, renal, adrenal, parapharyngeal, parotid, breast, muscle, ovarian, pancreatic, and cutaneous metastases [6, 7]. Isolated liver metastases are extremely rare, with very few cases reported with PTC and FTC [8–13] and only one case with HCTC [14].

Detection of distant metastatic disease at less common sites can be challenging in patients with HCTC. There have been various reports regarding the radioactive iodine (RAI) avidity of HCTC metastases, with RAI uptake 22.5–69% in different studies [1, 15–17]. In contrast, FDG-PET has a sensitivity of 92–96% and specificity of 80–95% [18], which would make it a more preferable option for detection of metastases when suspected based on Tg level. Other cross sectional imaging modalities such as CT and/or MRI are also helpful when the above imaging techniques are negative. This was particularly evident in our patient, as the initial PET-CT did not show evidence of metastatic disease, but subsequent abdominal CT 4 months later demonstrated the liver metastasis. However, a repeat PET/CT 1 month after the abdominal CT did show this lesion, which, along with the rising Tg, suggests that there was interval growth of the liver lesion allowing detectable FDG uptake.

The preferable treatment for most types of isolated liver metastases is surgical resection; however this may not be always feasible due to patient or tumor characteristics. In the past years, there have been significant breakthroughs in the treatment of liver metastatic disease, and multiple nonresection methods have been introduced. These include the thermal ablation techniques of radiofrequency ablation (RFA) and microwave ablation, which have replaced the older method of cryotherapy, and irreversible electroporation, which is a newer method and not as widely used [19]. Compared to RFA, MWA can heat the liver tissue more effectively, and when it is close to blood vessels, the temperature does not drop as much [19]. Both methods of thermal ablation have shown good results in the treatment of hepatic metastasis based on studies performed in patients with colorectal cancer. Hof et al. demonstrated that percutaneous RFA can be used as an alternative to surgical resection of liver metastases from colorectal cancer, with comparable overall survival [20]. Shibata et al. demonstrated that MWA is comparable to surgical resection in colorectal cancer [21]. Less data are available for noncolorectal cancers, but it seems that the rate of local recurrence is higher and the MWA procedure is most successful in tumors <3 cm [22].

There are scarce published data regarding thermal ablation of liver metastases of thyroid cancer origin. These are almost exclusively related to medullary thyroid cancer except for a case of rapidly progressive FTC metastasis, with RFA used mainly for cytoreduction and palliation [23, 24]. In addition, to our knowledge, there are no available published data regarding treatment of any distal thyroid cancer metastases with MWA. The reported patients with thyroid cancer and isolated liver metastases have been treated with surgical resection [8–10, 12] and radioactive iodine [11].

Laparoscopic MWA was chosen for this patient because the tumor was <3 cm, surgical resection would have required a major open procedure with significant morbidity, and there was a possibility that other metastases would arise in the future that may require additional procedures. For unresectable metastatic disease in patients with HCTC, tyrosine kinase inhibitors have also been used [25], but we believe that for this patient, given the absence of other metastases, MWA has the likelihood of cure with less potential toxicity. The patient's Tg is still detectable in the months following the procedure, which either enhances our original suspicion for microscopic disease at other sites or suggests local recurrence and requires close clinical follow-up. However, at this time, he has a biochemical incomplete response to therapy, which has increased his projected overall survival compared to his previous structural incomplete response to therapy.

In summary, we present a rare case of HCTC with isolated liver metastasis. To our knowledge, there has only been one case with HCTC with isolated liver metastasis described in the literature but none treated with microwave ablation. Endocrinologists and other physicians caring for patients with solitary or low volume liver metastatic disease should be aware of thermoablation, whether done by MWA or RFA and through either a percutaneous or a laparoscopic approach, as an alternative treatment to surgical resection for these patients.

## Figures and Tables

**Table 1:** 

Months after thyroidectomy	TSH (0.55–4.78 *µ*IU/mL)	Tg (1.6–50 ng/mL)
1	9.661	40
2	216.12	62 (stimulated)
8	0.332	318.1
10	Liver metastasis ablation	
11	0.036	0.6
19	0.159	1.3
22	0.033	1.9
